# The show goes on….

**DOI:** 10.4103/0019-5545.37308

**Published:** 2007

**Authors:** G. Swaminath

**Affiliations:** Department of Psychiatry, Dr. B. R. Ambedkar Medical College, Kadugondanahalli, Bangalore - 560045, India

Once you hear the details of the victory, it is hard to distinguish it from a defeat

- Jean-Paul Sartre

“Do not change horses midstream” is a popular adage. While this seems an ideal and necessary approach to follow when given a choice, occasionally these circumstances are thrust on you, all of a sudden, rendering coping difficult. It is a measure of the strength of the institution to be able to overcome this modification and carry on with its responsibilities.

A couple of months ago, the Indian Journal of Psychiatry (IJP) was affected by a transformation at the helm, due to nullification of the process of initial election. This “revolution” was deemed necessary by many vocal constituents who opined that a few initial pains would in time lead to long-term gain, i.e., “No pain, no gain.”

This led to a change in the editorial team of IJP, which has resulted in the delay in publication of this issue. Thanks, however, to the clear direction given to the editorial team by the executive committee of the Indian Psychiatric Society, as well as assistance to weather the flux created by the sudden transformation, the IJP is now in your hands, though late.

The change in the editorial team, which may seem dramatic, however has not led to much instability. I have now taken over as Editor in the interim period, and the previous editor Dr. T. S. Sathyanarayana Rao has been designated as the Chief Advisor to the IJP. There has been a reshuffle of posts but the collective responsibilities, which had earlier been shared, continue to be shared with the same intensity and vigor, with the sole aim of giving you a journal you would be proud of.

It is said that the best management is where there is no need for an administrator. Here systems and procedures would be duly laid down, and following these meticulously would lead to a good product. I compliment my predecessor and Chief Advisor Dr. T. S. Sathyanarayana Rao for having put in place these systems, making my task easier. I assure you that the next issue will be in your hands on time.

Dr. T. S. S. Rao, in the past issue of IJP 49(2), had put forth some enthusiastic proposals.[1] They included plans to archive all past journals since 1958 for electronic viewing; to bring out a commemorative volume during ANCIPS 2008 at Kolkata, a special issue of past presidential addresses; and to hold a special session and exhibition for the IJP at ANCIPS 2008; these are all still going to materialize, thanks to his energetic involvement and leadership. My many thanks to him!

On behalf of the organizers of the golden jubilee celebration of our journal, I request the involvement, help and guidance of all members to make it a memorable one.

Thanks to the effort of the editorial team under Dr. Rao, we have succeeded in indexing our journal in various indexing agencies. They are:

- SCOPUS
- DOAJ
- Index Copernicus
- Health and Wellness Research Center
- Health Reference Center Academic
- InfoTrac One File
- Expanded Academic ASAP
- Genamics JournalSeek
- Ulrich's International Periodical Directory
- EBSCO Publishing's Electronic Databases
- Google Scholar

Any ideas, suggestions, comments and criticisms could be directed to me through any means of communication – mobile, e-mail or snail mail. Contact me at editor@indianjpsychiatry.org or my personal e-mail swamyg@sify.com. Remember that brickbats have more chance of making into the journal (if written in the form of letters to editor) than bouquets, as they would generate many responses and hopefully enhance the quality of our journal.

I end by wishing you all happy reading!
